# Supplementary material for: Deep Immune Profiling of Multiple Myeloma at Diagnosis and under Lenalidomide Maintenance Therapy
Source: Cancers (Basel). 2023 May 4;15(9):2604. doi: 10.3390/cancers15092604 (PMC10177338; doi:10.3390/cancers15092604)
Supplement: Supplementary file 1 [file cancers-15-02604-s001.zip › Supplementary.pdf]

Supplementary Information

Deep immune profiling of multiple myeloma at diagnosis and under lenalidomide maintenance therapy

Sini Luoma [1†,*], Philipp Sergeev [2,†], Komal Kumar Javarappa[2,] Tiina J Öhman [3], Markku Varjosalo [3], Marjaana Säily [4], Pekka Anttila [1], Marja Sankelo [5], Anu Partanen [6], Anne Nihtinen [7], Caroline A. Heckman [2,#] and Raija Silvennoinen [1,6,#]

Supplementary methods

T cell exhaustion by flow cytometry

Freshly thawed BMMNCs were washed with staining buffer (SB: 2% fetal bovine serum in PBS) and incubated with DNAse I (Promega, Madison WI) for 30 minutes at +37°C. Afterwards, the cells were plated at $1x10^5$ cells per well in a 96-well plate. The cells were suspended in 25 μL of SB with the following antibodies: CD4-BV510 (1:50) (BD Biosciences, San Jose, CA, #566804), CD8-APC (1:50), TIM-3-BB515 (1:25) (BD Biosciences, #565569),

LAG-3-PE (1:25) (BD Biosciences, #565617), PD-1-PE-CF594 (1:25) (BD Biosciences, #565024), NKG2a-BV605 (BD Biosciences, #74921) (1:50), and TIGIT-BV-421 (1:50) (BD Biosciences, #747844). Antibody concentrations were optimized prior to staining. After 30 minutes of incubation at RT, the cells were washed with SB and resuspended in 25 μL of Annexin V binding buffer containing 7-AAD (BD Biosciences, San Diego, CA). Flow cytometry was performed on an iQue Screener Plus instrument (Sartorius, Göttingen, Germany) and the data analyzed with FlowJo V10 software (BD Life Sciences, Ashland, OR).

Manual gating

The normalized flow cytometry standard files (FCS) were imported to FlowJo and Cytobank (Cytobank Inc., Santa Clara, CA) environments for further analysis. Two-dimensional manual gating was performed to identify specific populations. Prior to the identification of immune cell populations, a cleanup gating strategy was implemented to exclude debris, beads, doublets, dead cells and unwanted events from the analysis. For that we used common (DNA intercalator, bead, event length, and cell viability) and Gaussian (center, width, offset, and residuals) parameters for every event (Figure S1) (1). After the data preprocessing, we utilized live cells ($Ir191^+/Ir193^+$ $103Rh^-$) for further analysis to define all the immune cell populations based on the expression of the surface markers, using Maxpar Direct immune Profiling Assay gating strategy (2). Along with this gating strategy, we introduced several additional gatings, based on literature findings and unsupervised clustering approaches (see below) (Table S2).

Supplementary tables

Table S1. Maxpar Direct Immune Profiling Assay Panel

Antibody	Clone	Mass
CD45	HI30	89Y
CCR6 (CD196)	G034E3	141Pr
CD123	6H6	143Nd
CD19	HIB19	144Nd
CD4	RPA-T4	145Nd
CD8a	RPA-T8	146Nd
CD11c	Bu15	147Sm
CD16	3G8	148Nd
CD45RO	UCHL1	149Sm
CD45RA	HI100	150Nd
CD161	HP-3G10	151Eu
CCR4 (CD194)	L291H4	152Sm
CD25	BC96	153Eu
CD27	O323	154Sm
CD57	HCD57	155Gd
CXCR3 (CD183)	G025H7	156Gd
CXCR5 (CD185)	J252D4	158Gd
CD28	CD28.2	160Gd
CD38	HB-7	161Dy
NCAM (CD56)	NCAM16.2	163Dy
TCRγδ	B1	164Dy
CD294	BM16	166Er
CCR7 (CD197)	G043H7	167Er
CD14	63D3	168Er
CD3	UCHT1	170Er
CD20	2H7	171Yb
CD66b	G10F5	172Yb
HLA-DR	LN3	173Yb
IgD	IA6-2	174Yb
CD127	A019D5	176Yb
Cell-ID	Intercalator	103Rh

Table S2. Gating strategy and abundance of immune populations of bone marrow

Table is provided as separate spreadsheet file

Table S3. Cytogenetics information and BM sample collection timepoints.

Table is provided as separate spreadsheet file

Table S4. Median raw marker expression values for different cohorts of patients

Table is provided as separate spreadsheet file

Table S5. Median population abundances, based on UMAP clustering and gating.

Table is provided as separate spreadsheet file

Supplementary figures

Figure S1. Cells clean up strategy. Bi-axial dot plots represent the order of cell clean up from beads and cells of low quality/viability. Boxes show the percentage of cells included in the gate relative to the parental population.

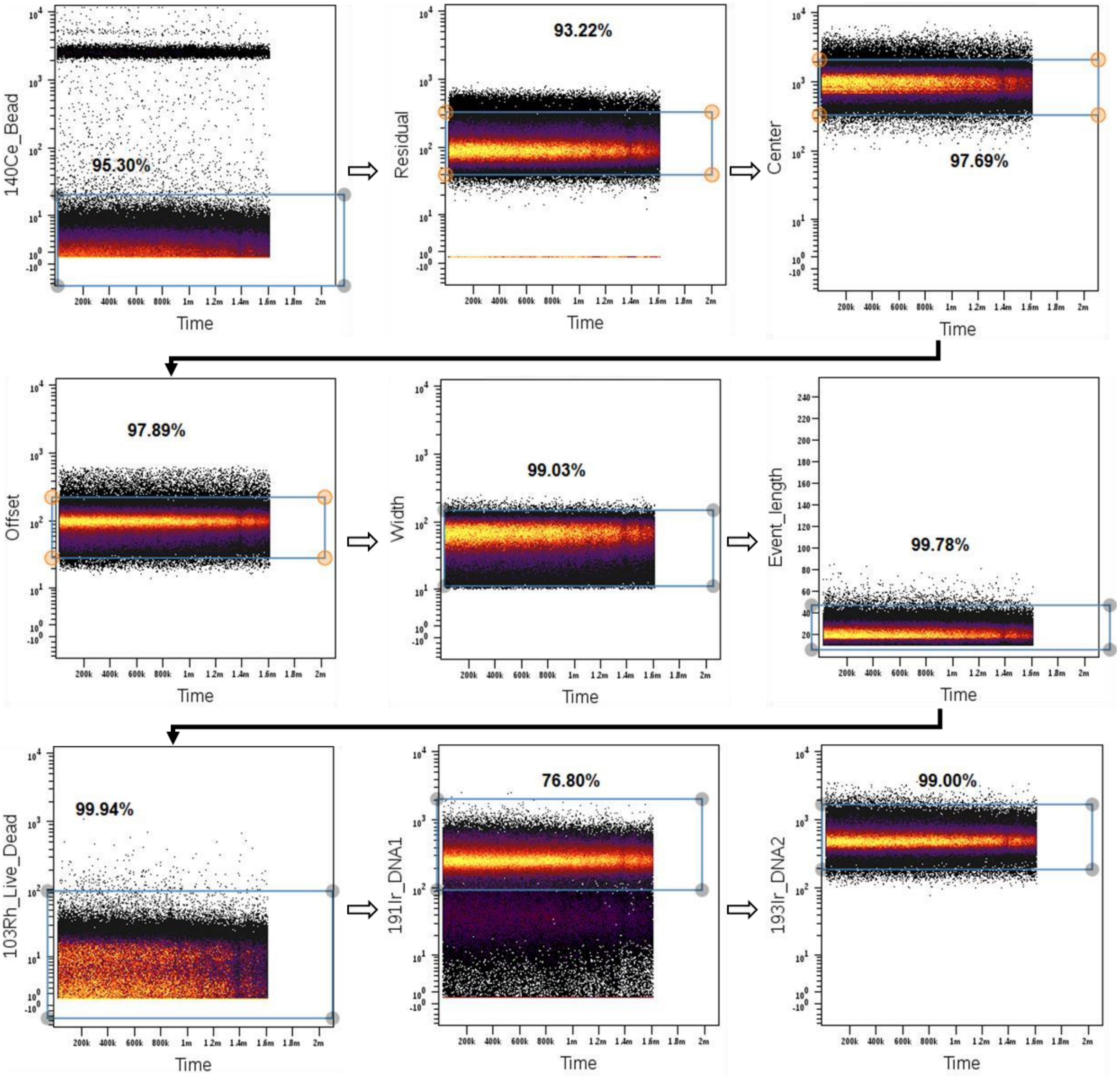

Figure S2. **Phenotype of metaclusters of non-malignant cells in different groups of patients.** The log1p-transformed, scaled and centered median values of expression intensity are used for the depicting of the intensity of the expression

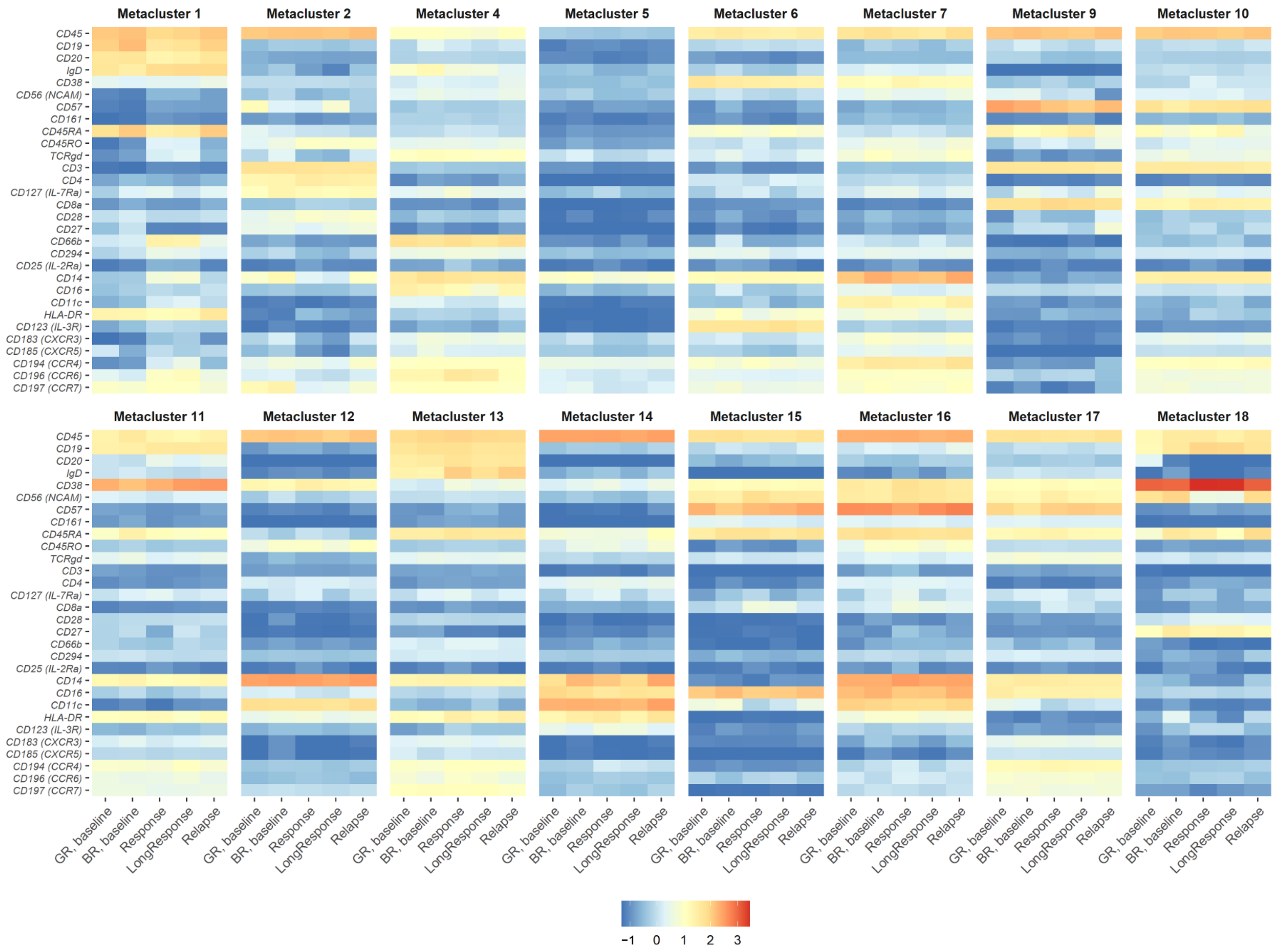

Figure S3. **T and NK cells' exhaustion markers.** Box plots show median values of population abundance with hinges at the 25th and the 75th percentiles and whiskers. NK cells were considered to be a part of the CD4−CD8− population, which was therefore used to track changes in NK cells.

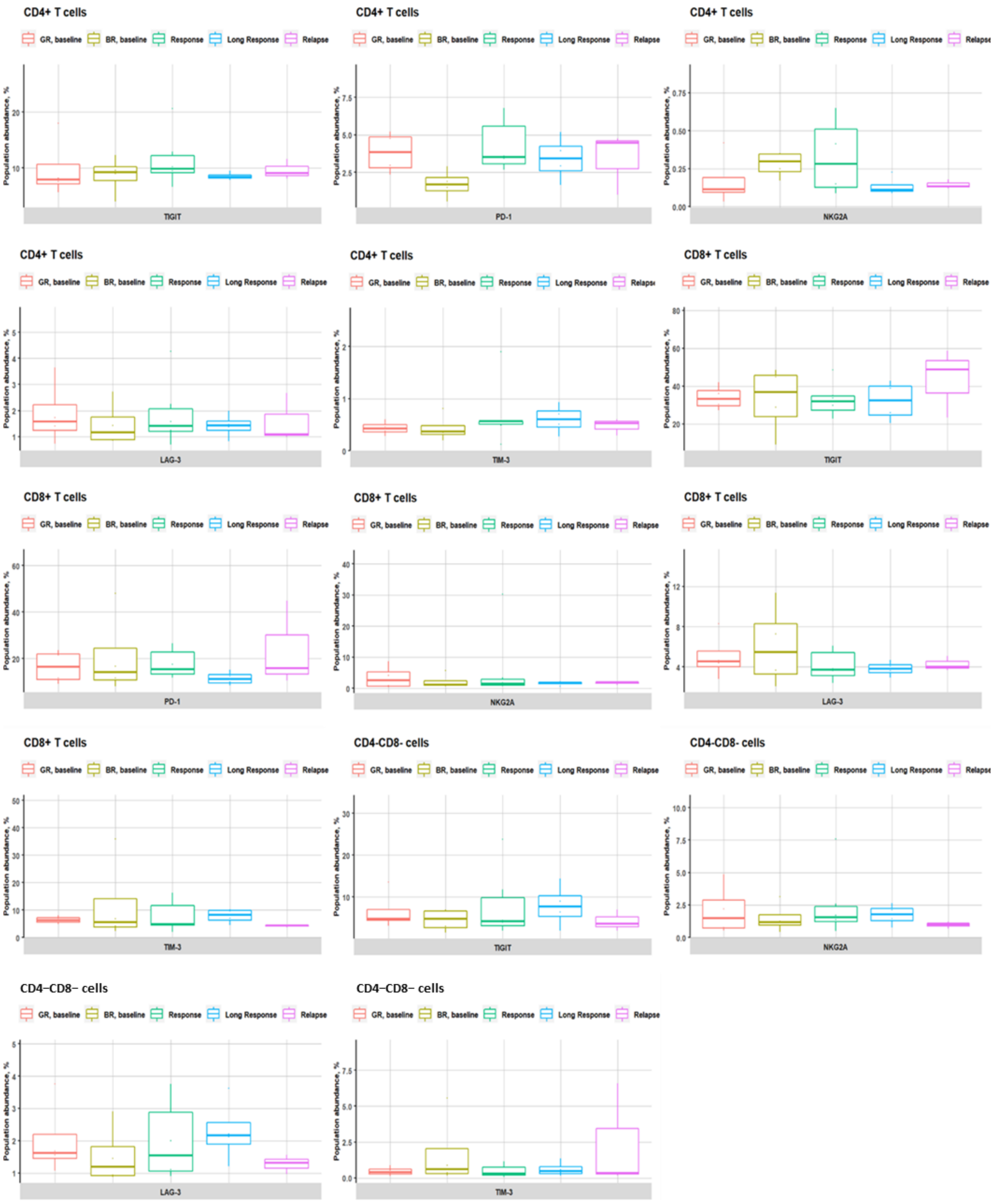

Figure S4. Comparative analysis of differences between high and standard risk samples at the baseline and at response/relapse. The median abundance with 25th and 75th percentiles and whiskers of populations in A) baseline and B) response/relapse cohorts are shown.

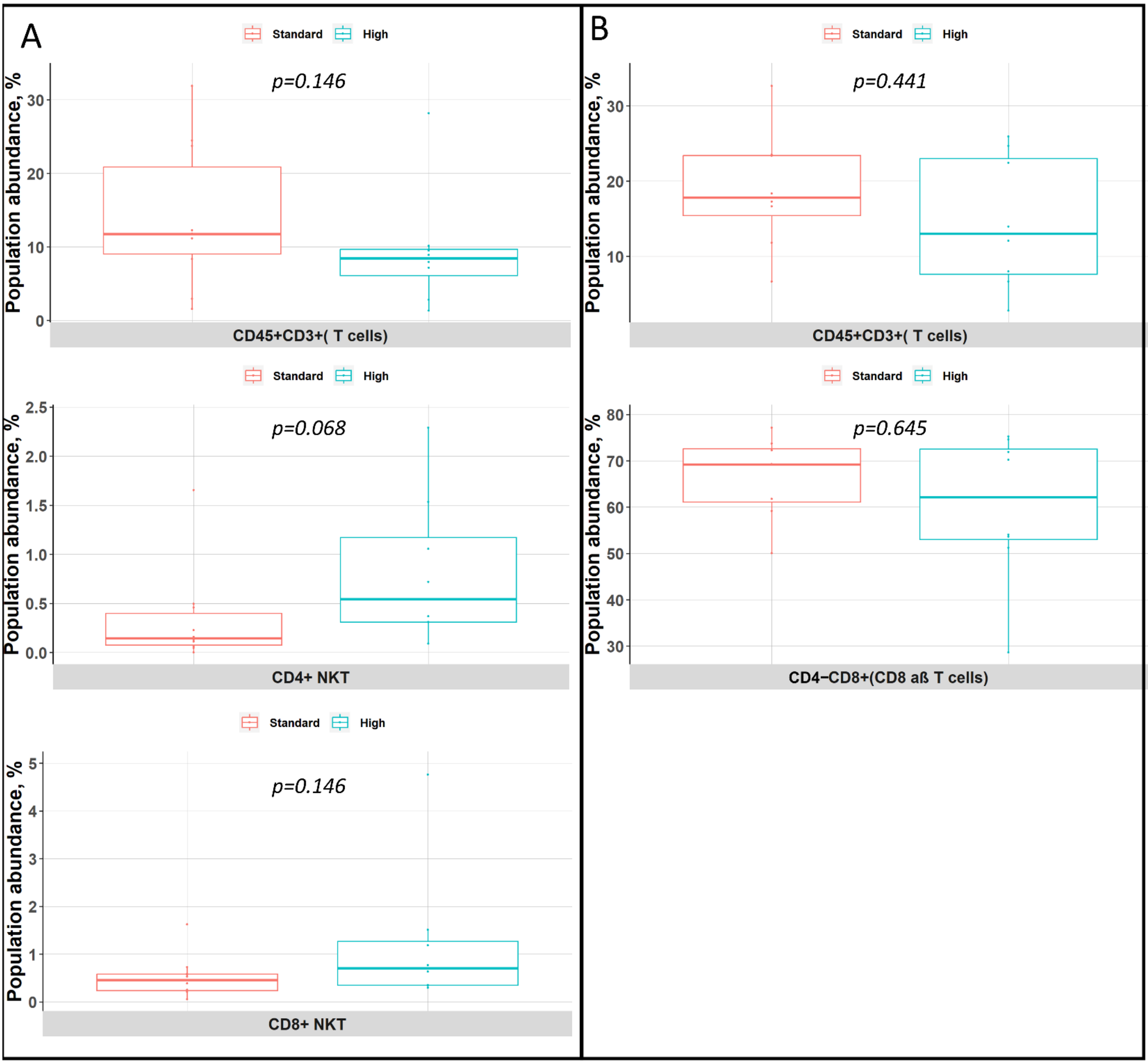

Figure S5. Longitudinal analysis of bad responders at different stages. A-D) The boxplots with median values, hinges and whiskers are represented. The values of the same patient are linked with the line. If the ratio between two groups was in the range from 0.9 to 1.1, it was depicted as “No changes”. The dashed line represents the median value of the populations’ abundance of the earlier time point on the plot. Circles indicate samples with high cytogenetic risk.

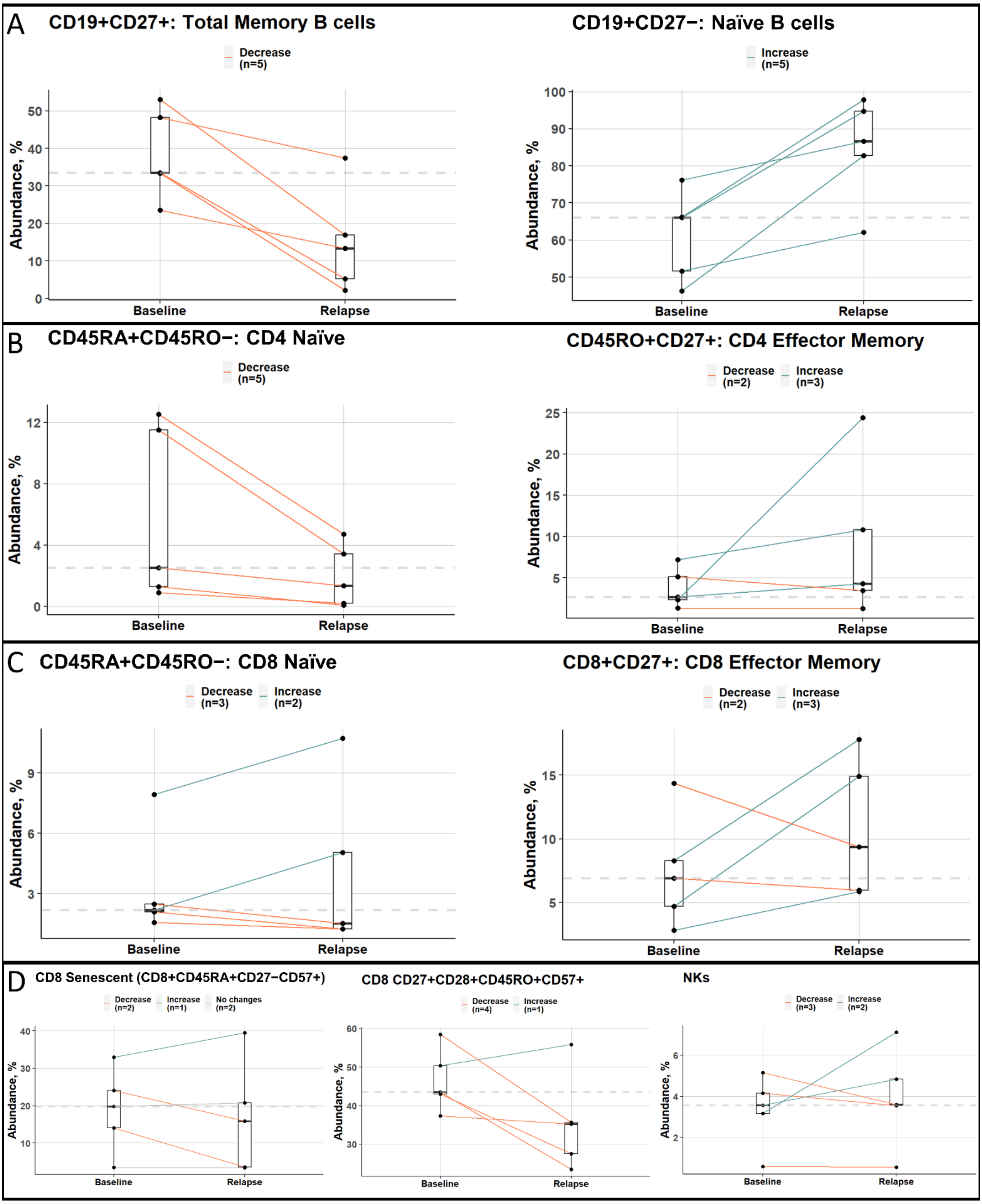

Supplementary Reference list

1. Olsen LR, Leipold MD, Pedersen CB, Maecker HT. The anatomy of single cell mass cytometry data. Cytom Part A [Internet]. 2019 Feb 2;95(2):156–72. Available from: https://onlinelibrary.wiley.com/doi/10.1002/cyto.a.23621

2. Bagwell CB, Hunsberger B, Hill B, Herbert D, Bray C, Selvanantham T, et al. Multi‐site reproducibility of a human immunophenotyping assay in whole blood and peripheral blood mononuclear cells preparations using CyTOF technology coupled with Maxpar Pathsetter, an automated data analysis system. Cytom Part B Clin Cytom [Internet]. 2020 Mar;98(2):146–60. Available from: https://onlinelibrary.wiley.com/doi/10.1002/cyto.b.21858
